# Supplementary material for: Some Interventions to Shift Meta-Norms Are Effective for Changing Behaviors in Low- and Middle-Income Countries: A Rapid Systematic Review
Source: Int J Environ Res Public Health. 2022 Jun 14;19(12):7312. doi: 10.3390/ijerph19127312 (PMC9223853; doi:10.3390/ijerph19127312)
Supplement: Supplementary file 1 [file ijerph-19-07312-s001.zip › ijerph-1727716-supplementary.pdf]

Review

Some Interventions to Shift Meta-Norms Are Effective for Changing Behaviors in Low- and Middle-Income Countries: A Rapid Systematic Review

Supplementary Materials

Annette N. Brown

FHI 360, Washington, DC 20009, USA; abrown@fhi360.org

Figure S1. PRISMA 2009 Checklist.

Section/Topic	#	Checklist Item	Reported on Page #
**TITLE**			
Title	1	Identify the report as a systematic review, meta-analysis, or both.	1
**ABSTRACT**			
Structured summary	2	Provide a structured summary including, as applicable: background; objectives; data sources; study eligibility criteria, participants, and interventions; study appraisal and synthesis methods; results; limitations; conclusions and implications of key findings; systematic review registration number.	1
**INTRODUCTION**			
Rationale	3	Describe the rationale for the review in the context of what is already known.	1-2
Objectives	4	Provide an explicit statement of questions being addressed with reference to participants, interventions, comparisons, outcomes, and study design (PICOS).	3-5
**METHODS**			
Protocol and registration	5	Indicate if a review protocol exists, if and where it can be accessed (e.g., Web address), and, if available, provide registration information including registration number.	3
Eligibility criteria	6	Specify study characteristics (e.g., PICOS, length of follow-up) and report characteristics (e.g., years considered, language, publication status) used as criteria for eligibility, giving rationale.	4-5
Information sources	7	Describe all information sources (e.g., databases with dates of coverage, contact with study authors to identify additional studies) in the search and date last searched.	5-6
Search	8	Present full electronic search strategy for at least one database, including any limits used, such that it could be repeated.	Figure 1
Study selection	9	State the process for selecting studies (i.e., screening, eligibility, included in systematic review, and, if applicable, included in the meta-analysis).	6
Data collection process	10	Describe method of data extraction from reports (e.g., piloted forms, independently, in duplicate) and any processes for obtaining and confirming data from investigators.	6-7
Data items	11	List and define all variables for which data were sought (e.g., PICOS, funding sources) and any assumptions and simplifications made.	6-7

Risk of bias in individual studies	12	Describe methods used for assessing risk of bias of individual studies (including specification of whether this was done at the study or outcome level), and how this information is to be used in any data synthesis.	7
Summary measures	13	State the principal summary measures (e.g., risk ratio, difference in means).	N/A
Synthesis of results	14	Describe the methods of handling data and combining results of studies, if done, including measures of consistency (e.g., I^2) for each meta-analysis.	N/A
Risk of bias across studies	15	Specify any assessment of risk of bias that may affect the cumulative evidence (e.g., publication bias, selective reporting within studies).	N/A
Additional analyses	16	Describe methods of additional analyses (e.g., sensitivity or subgroup analyses, meta-regression), if done, indicating which were pre-specified.	N/A
**RESULTS**			
Study selection	17	Give numbers of studies screened, assessed for eligibility, and included in the review, with reasons for exclusions at each stage, ideally with a flow diagram.	8<br>Fig. 2
Study characteristics	18	For each study, present characteristics for which data were extracted (e.g., study size, PICOS, follow-up period) and provide the citations.	11-15
Risk of bias within studies	19	Present data on risk of bias of each study and, if available, any outcome level assessment (see item 12).	16-17
Results of individual studies	20	For all outcomes considered (benefits or harms), present, for each study: (a) simple summary data for each intervention group (b) effect estimates and confidence intervals, ideally with a forest plot.	19-23
Synthesis of results	21	Present results of each meta-analysis done, including confidence intervals and measures of consistency.	N/A
Risk of bias across studies	22	Present results of any assessment of risk of bias across studies (see Item 15).	N/A
Additional analysis	23	Give results of additional analyses, if done (e.g., sensitivity or subgroup analyses, meta-regression [see Item 16]).	N/A
**DISCUSSION**			
Summary of evidence	24	Summarize the main findings including the strength of evidence for each main outcome; consider their relevance to key groups (e.g., healthcare providers, users, and policy makers).	25-26

Limitations	25	Discuss limitations at study and outcome level (e.g., risk of bias), and at review-level (e.g., incomplete retrieval of identified research, reporting bias).	27
Conclusions	26	Provide a general interpretation of the results in the context of other evidence, and implications for future research.	27
**FUNDING**			
Funding	27	Describe sources of funding for the systematic review and other support (e.g., supply of data); role of funders for the systematic review.	28

From: Moher D, Liberati A, Tetzlaff J, Altman DG, The PRISMA Group (2009). Preferred Reporting Items for Systematic Reviews and Meta-Analyses: The PRISMA Statement. PLoS Med 6(7): e1000097. doi:10.1371/journal.pmed1000097

For more information, visit: **www.prisma-statement.org**.

Table S1. Index searches.

Platform and Indexes	Search Hits	Search Strategy
EBSCO Host (primary studies)<br><br>Academic Search Premier<br>EconLit<br>Education Full Text (H.W. Wilson)<br>Environment Complete<br>ERIC<br>Global Health<br>APA PsycInfo	449	In title/abstract/keywords: (norms AND (intervention OR interventions OR program* OR project OR projects OR policy OR policies OR experiment OR experiments) AND (behavior OR behaviour) AND (change OR improv* OR impact OR increas* OR decreas* OR effect) AND (measur* OR estimat* OR evaluat* OR trial OR test OR experiment) AND ("developing country" OR "developing countries" OR "low resource country" OR "low resource countries" OR "low-resource country" OR "low-resource countries" OR "low income country" OR "low income countries" OR "low-income country" OR "low-income countries" OR "middle income country" OR "middle income countries" OR "middle-income country" OR "middle-income countries" OR "low- and middle-income countries" OR "low and middle income countries" OR LMIC OR LMICs OR "less-developed countries" OR "less developed countries" OR "resource limited countries" OR "resource-limited countries" OR "limited-resource countries" OR "limited resource countries" OR Afghanistan OR Albania OR Algeria OR Angola OR Argentina OR Armenia OR Azerbaijan OR Bangladesh OR Benin OR Belarus OR Belize OR Bhutan OR Bolivia OR Bosnia OR Herzegovina OR Botswana OR Brazil OR Bulgaria OR "Burkina Faso" OR Burundi OR Cambodia OR Cameroon OR "Cape Verde" OR "Cabo Verde" OR "Central African Republic" OR Chad OR China OR Colombia OR Comoros OR Congo OR "Costa Rica" OR "Cote d'Ivoire" OR "Ivory Coast" OR Cuba OR Djibouti OR Dominica OR "Dominican Republic" OR "East Timor" OR "Timor Leste" OR Ecuador OR Egypt OR "El Salvador" OR Eritrea OR Eswatini OR "Equatorial Guinea" OR Ethiopia OR Fiji OR Gabon OR Gambia OR Gaza OR Georgia OR Ghana OR Grenada OR Guatemala OR Guinea OR Guinea-Bissau OR Guyana OR Haiti OR Honduras OR India OR Maldives OR Indonesia OR Iran OR Iraq OR Jamaica OR Jordan OR Kazakhstan OR Kenya OR Kiribati OR Korea OR Kosovo OR "Kyrgyz Republic" OR "Lao PDR" OR Lebanon OR Lesotho OR Liberia OR Libya OR Macedonia OR Madagascar OR Malaysia OR Malawi OR Maldives OR Mali OR "Marshall Islands" OR Mauritania OR Mexico OR Micronesia OR Moldova OR Mongolia OR Montenegro OR Morocco OR Mozambique OR Myanmar OR Namibia OR Nepal OR Nicaragua OR Niger OR Nigeria OR Pakistan OR "Papua New Guinea" OR Paraguay OR Peru OR Philippines OR Russia OR "Russian Federation" OR Rwanda OR "Saint Lucia" OR "St Lucia" OR "Saint Vincent" OR "St Vincent" OR Grenadines OR Samoa OR "Sao Tome and Principe" OR Senegal OR Serbia OR Montenegro OR "Sierra Leone" OR "Sri Lanka" OR "Solomon Islands" OR Somalia OR "South Africa" OR Sudan OR Suriname OR Swaziland OR Syria OR "Syrian Arab Republic" OR Tajikistan OR Tanzania OR Thailand OR Togo OR Tonga OR Tunisia OR Turkey OR Turkmenistan OR Tuvalu OR Uganda OR Ukraine OR Uzbekistan OR Vanuatu OR Venezuela OR Vietnam OR "Viet Nam" OR "West Bank" OR Yemen OR Zambia OR Zimbabwe OR "South Asia" OR "Sub-Saharan Africa" OR "Sub Saharan Africa" OR "Subsaharan Africa" OR "Latin America" OR Carribean OR "East Asia" OR "Middle East" OR "North Africa" OR "Central Asia"))

		Limits: 2010-present<br>Source Type=Academic Journals, Journals, Reports, Working Papers (Excludes Books, Conference Materials, Dissertations, Magazines)
EBSCO Host (systematic reviews)<br><br>Academic Search Premier<br>EconLit<br>Education Full Text (H.W. Wilson)<br>Environment Complete<br>ERIC<br>Global Health<br>APA PsycInfo	38	In title/abstract/keywords: ((norms) AND (intervention OR interventions OR program* OR project OR projects OR policy OR policies) AND (behavior OR behaviour) AND (change OR improv* OR impact) AND (“systematic review” OR meta-analysis OR metaanalysis OR “evidence map”) AND (measure OR effect OR outcome OR indicator))<br><br>Limits: 2010-present<br>Source Type=Academic Journals, Journals, Reports, Working Papers (Excludes Books, Conference Materials, Dissertations, Magazines)
Scopus (primary studies)	969	In title/abstract/keywords: (norms AND (intervention OR interventions OR program* OR project OR projects OR policy OR policies OR experiment OR experiments) AND (behavior OR behaviour) AND (change OR improv* OR impact OR increas* OR decreas* OR effect) AND (measur* OR estimat* OR evaluat* OR trial OR test OR experiment) AND ("developing country" OR "developing countries" OR "low resource country" OR "low resource countries" OR "low-resource country" OR "low-resource countries" OR "low income country" OR "low income countries" OR "low-income country" OR "low-income countries" OR "middle income country" OR "middle income countries" OR "middle-income country" OR "middle-income countries" OR "low- and middle-income countries" OR "low and middle income countries" OR LMIC OR LMICs OR "less-developed countries" OR "less developed countries" OR "resource limited countries" OR "resource-limited countries" OR "limited-resource countries" OR "limited resource countries" OR Afghanistan OR Albania OR Algeria OR Angola OR Argentina OR Armenia OR Azerbaijan OR Bangladesh OR Benin OR Belarus OR Belize OR Bhutan OR Bolivia OR Bosnia OR Herzegovina OR Botswana OR Brazil OR Bulgaria OR "Burkina Faso" OR Burundi OR Cambodia OR Cameroon OR "Cape Verde" OR "Cabo Verde" OR "Central African Republic" OR Chad OR China OR Colombia OR Comoros OR Congo OR "Costa Rica" OR "Cote d'Ivoire" OR "Ivory Coast" OR Cuba OR Djibouti OR Dominica OR "Dominican Republic" OR "East Timor" OR "Timor Leste" OR Ecuador OR Egypt OR

		"El Salvador" OR Eritrea OR Eswatini OR "Equatorial Guinea" OR Ethiopia OR Fiji OR Gabon OR Gambia OR Gaza OR Georgia OR Ghana OR Grenada OR Guatemala OR Guinea OR Guinea-Bissau OR Guyana OR Haiti OR Honduras OR India OR Maldives OR Indonesia OR Iran OR Iraq OR Jamaica OR Jordan OR Kazakhstan OR Kenya OR Kiribati OR Korea OR Kosovo OR "Kyrgyz Republic" OR "Lao PDR" OR Lebanon OR Lesotho OR Liberia OR Libya OR Macedonia OR Madagascar OR Malaysia OR Malawi OR Maldives OR Mali OR "Marshall Islands" OR Mauritania OR Mexico OR Micronesia OR Moldova OR Mongolia OR Montenegro OR Morocco OR Mozambique OR Myanmar OR Namibia OR Nepal OR Nicaragua OR Niger OR Nigeria OR Pakistan OR "Papua New Guinea" OR Paraguay OR Peru OR Philippines OR Russia OR "Russian Federation" OR Rwanda OR "Saint Lucia" OR "St Lucia" OR "Saint Vincent" OR "St Vincent" OR Grenadines OR Samoa OR "Sao Tome and Principe" OR Senegal OR Serbia OR Montenegro OR "Sierra Leone" OR "Sri Lanka" OR "Solomon Islands" OR Somalia OR "South Africa" OR Sudan OR Suriname OR Swaziland OR Syria OR "Syrian Arab Republic" OR Tajikistan OR Tanzania OR Thailand OR Togo OR Tonga OR Tunisia OR Turkey OR Turkmenistan OR Tuvalu OR Uganda OR Ukraine OR Uzbekistan OR Vanuatu OR Venezuela OR Vietnam OR "Viet Nam" OR "West Bank" OR Yemen OR Zambia OR Zimbabwe OR "South Asia" OR "Sub-Saharan Africa" OR "Sub Saharan Africa" OR "Subsaharan Africa" OR "Latin America" OR Carribean OR "East Asia" OR "Middle East" OR "North Africa" OR "Central Asia"))<br><br>Limits: 2010-present<br>Document Type=Article, Review (Excludes Conference Paper, Book Chapter, Conference Review, Letter, Editorial, Erratum)
Scopus (systematic reviews)	96	In title/abstract/keywords: ((norms) AND (intervention OR interventions OR program* OR project OR projects OR policy OR policies) AND (behavior OR behaviour) AND (change OR improv* OR impact) AND (“systematic review” OR meta-analysis OR metaanalysis OR “evidence map”) AND (measure OR effect OR outcome OR indicator))<br><br>Limits: 2010-present<br>Document Type=Article, Review (Excludes Conference Paper, Book Chapter, Conference Review, Letter, Editorial, Erratum)
Pubmed (both)	255	("Social Norms"[MeSH] OR "social norms"[tiab]) AND (intervention OR program) AND (evaluation OR trial OR "systematic review" OR meta-analysis) AND ("developing country" OR "developing countries" OR "low resource country" OR "low resource countries" OR "low-resource country" OR "low-resource countries" OR "low income country" OR "low income countries" OR "low-income country" OR "low-income countries" OR "middle income country" OR "middle income countries" OR "middle-income country" OR "middle-income countries" OR "low- and middle-income countries" OR "low and middle income countries" OR LMIC OR LMICs

		OR "less-developed countries" OR "less developed countries" OR "resource limited countries" OR "resource-limited countries" OR "limited-resource countries" OR "limited resource countries" OR Afghanistan OR Albania OR Algeria OR Angola OR Argentina OR Armenia OR Azerbaijan OR Bangladesh OR Benin OR Belarus OR Belize OR Bhutan OR Bolivia OR Bosnia OR Herzegovina OR Botswana OR Brazil OR Bulgaria OR "Burkina Faso" OR Burundi OR Cambodia OR Cameroon OR "Cape Verde" OR "Cabo Verde" OR "Central African Republic" OR Chad OR China OR Colombia OR Comoros OR Congo OR "Costa Rica" OR "Cote d'Ivoire" OR "Ivory Coast" OR Cuba OR Djibouti OR Dominica OR "Dominican Republic" OR "East Timor" OR "Timor Leste" OR Ecuador OR Egypt OR "El Salvador" OR Eritrea OR Eswatini OR "Equatorial Guinea" OR Ethiopia OR Fiji OR Gabon OR Gambia OR Gaza OR Georgia OR Ghana OR Grenada OR Guatemala OR Guinea OR Guinea-Bissau OR Guyana OR Haiti OR Honduras OR India OR Maldives OR Indonesia OR Iran OR Iraq OR Jamaica OR Jordan OR Kazakhstan OR Kenya OR Kiribati OR Korea OR Kosovo OR "Kyrgyz Republic" OR "Lao PDR" OR Lebanon OR Lesotho OR Liberia OR Libya OR Macedonia OR Madagascar OR Malaysia OR Malawi OR Maldives OR Mali OR "Marshall Islands" OR Mauritania OR Mexico OR Micronesia OR Moldova OR Mongolia OR Montenegro OR Morocco OR Mozambique OR Myanmar OR Namibia OR Nepal OR Nicaragua OR Niger OR Nigeria OR Pakistan OR "Papua New Guinea" OR Paraguay OR Peru OR Philippines OR Russia OR "Russian Federation" OR Rwanda OR "Saint Lucia" OR "St Lucia" OR "Saint Vincent" OR "St Vincent" OR Grenadines OR Samoa OR "Sao Tome and Principe" OR Senegal OR Serbia OR Montenegro OR "Sierra Leone" OR "Sri Lanka" OR "Solomon Islands" OR Somalia OR "South Africa" OR Sudan OR Suriname OR Swaziland OR Syria OR "Syrian Arab Republic" OR Tajikistan OR Tanzania OR Thailand OR Togo OR Tonga OR Tunisia OR Turkey OR Turkmenistan OR Tuvalu OR Uganda OR Ukraine OR Uzbekistan OR Vanuatu OR Venezuela OR Vietnam OR "Viet Nam" OR "West Bank" OR Yemen OR Zambia OR Zimbabwe OR "South Asia" OR "Sub-Saharan Africa" OR "Sub Saharan Africa" OR "Subsaharan Africa" OR "Latin America" OR Carribean OR "East Asia" OR "Middle East" OR "North Africa" OR "Central Asia")<br><br>Limits: 2010-present

Table S2. Website searches.

Website / URL	Search Hits	Search Strategy
Cochrane Library / https://www.cochranelibrary.com/advanced-search?cookiesEnabled	19	Title Abstract Keyword = norms
3ie Evidence Portal / https://www.developmentevidence.3ieimpact.org	101	Search terms = norms; Year of publication = 2010 - 2020
Campbell / https://www.campbellcollaboration.org/website-search?searchword=work	27	Keyword = behavior change
J-PAL / https://www.povertyactionlab.org/evaluations	76	Search terms = "behavior change" norms; Status = completed
IPA / http://www.poverty-action.org/publications	34	Type = published paper, working; Topics = behavioral design
IRC / https://www.rescue.org/reports-and-resources	14	Search terms = norms
IFPRI / https://www.ifpri.org/publications	15	Search terms = norms; Type Of = journal article
World Bank / https://www.worldbank.org/en/research	44	Search terms = "norms" "behavior change"
